# Gastrointestinal (GI)-Tract Microbiome Derived Neurotoxins and their Potential Contribution to Inflammatory Neurodegeneration in Alzheimer’s Disease (AD)

**Published:** 2021-05-25

**Authors:** Walter J. Lukiw, Lisa Arceneaux, Wenhong Li, Taylor Bond, Yuhai Zhao

**Affiliations:** 1 LSU Neuroscience Center, Louisiana State University Health Sciences Center, New Orleans, LA, United States; 2 Department of Ophthalmology, LSU Health Sciences Center, New Orleans, LA, United States; 3 Department of Neurology, Louisiana State University Health Sciences Center, New Orleans, LA, United States; 4 Department of Pharmacology, School of Pharmacy, Jiangxi University of Traditional Chinese Medicine (TCM), Nanchang, China; 5 Department of Anatomy and Cell Biology, Louisiana State University, New Orleans, LA, United States

**Keywords:** Aging, Alzheimer’s disease, *Bacteroides fragilis*, Brain microbiome, Dysbiosis, Lipopolysaccharide, microRNA-146a, miRNA-155, Necrobiome, NF-kB, Reactive oxygen species, Thanatomicrobiome

## Abstract

The human gastrointestinal (GI)-tract microbiome is a rich, complex and dynamic source of microorganisms that possess a staggering diversity and complexity. Importantly there is a significant variability in microbial complexity even amongst healthy individuals-this has made it difficult to link specific microbial abundance patterns with age-related neurological disease. GI-tract commensal microorganisms are generally beneficial to human metabolism and immunity, however enterotoxigenic forms of microbes possess significant potential to secrete what are amongst the most neurotoxic and pro-inflammatory biopolymers known. These include toxic glycolipids such as lipopolysaccharide (LPS), enterotoxins, microbial-derived amyloids and small non-coding RNA. One major microbial species of the GI-tract microbiome, about ~100-fold more abundant than *Escherichia coli* in deep GI-tract regions is *Bacteroides fragilis*, an anaerobic, rod-shaped Gram-negative bacterium. *B. fragilis* can secrete: (i) a particularly potent, pro-inflammatory and unique LPS subtype (BF-LPS); and (ii) a zinc-metalloproteinase known as *B. fragilis*-toxin (BFT) or *fragilysin*. Ongoing studies indicate that BF-LPS and/or BFT disrupt paracellular-and transcellular-barriers by cleavage of intercellular-proteins resulting in ‘leaky’ barriers. These barriers: (i) become defective and more penetrable with aging and disease; and (ii) permit entry of microbiome-derived neurotoxins into the systemic-circulation from which they next transit the blood-brain barrier and gain access to the CNS. Here LPS accumulates and significantly alters homeostatic patterns of gene expression. The affinity of LPS for neuronal nuclei is significantly enhanced in the presence of amyloid beta 42 (Aβ42) peptides. Recent research on the appearance of the brain thanatomicrobiome at the time of death and the increasing likelihood of a complex brain microbiome are reviewed and discussed. This paper will also highlight some recent advances in this extraordinary research area that links the pro-inflammatory exudates of the GI-tract microbiome with innate-immune disturbances and inflammatory-signaling within the CNS with reference to Alzheimer’s disease (AD) wherever possible.

## Introduction and Overview

### The human GI-tract microbiome

Consisting of about ~10^14^ microbes the gastrointestinal (GI)-tract microbiome comprises the largest repository of microorganisms anywhere in the human body. The unified human gastrointestinal genome (UHGG) collection has recently classified 204,938 non-redundant genomes from 4,644 GI-tract prokaryotes that encode >170 million protein sequences [1]. About ~98% of this microbiome are made up of several thousand genetically diverse bacterial species that form a complex, dynamic, and highly interactive microbial community [1-3]. While the GI-tract microbiome consists chiefly of aerobic and anaerobic bacteria, archaebacteria, fungi, protozoa, viruses and other microorganisms make up the remainder [4-7]. In general, this abundant, diverse and complex array of commensal microorganisms, with the highest known density of microbes found anywhere in nature (approaching ~10^11^ microbes per gm of stool [8]; contribute to the digestive, metabolic, nutritional and immune health of the host including protection against foreign microbial pathogens. However under non-homeostatic conditions or when stressed, or with aging and disease, or with diets low in fiber and high in fat and cholesterol these same microorganisms are capable of becoming dangerous ‘enterotoxigenic’ microbes generating some of the most potent pro-inflammatory neurotoxins known [7,9-16]; see below). There has recently been an expanding recognition of the ability of neurotoxins released by GI-tract resident microbes to influence metabolic and neuro-immune functions in health and disease well beyond the confines of an intact GI-tract [7,12,13,16,17]. As humans age, senescence of the GI-tract barrier integrity and immune system appears to result in an increased systemic microbial and/or microbial-derived neurotoxin burden as host-cell mediated biophysical barriers become “leaky” and innate-immune responses progressively decline. Maintaining the fidelity and integrity of biophysical barriers-including, prominently, the GI-tract barrier, and the blood-brain barrier (BBB)-against an increasing microbial pressure is essential to exclude an increasing bacterial load, in keeping GI-tract-derived neurotoxins like LPS and other microbial toxins out of the portal and/or systemic circulation, and in minimizing neurotoxin access to the brain and CNS compartments. In fact GI-tract microbiome-derived neurotoxin entry into the systemic circulation via leaky biophysical barriers and the induction of ’systemic inflammation’: (i) appears to be a precursor event to the onset of inflammatory neurodegeneration in the brain and CNS [7,18-20]; and (ii) an important step in establishing pathways of pathological communication between the GI-tract, the systemic circulation and central innate-immune systems involved in progressive age-related neurological disease [17,18,21-24]. Multiple independent reports support the observation of early GI-tract disruption, increased pro-inflammatory neurotoxins in the systemic circulation and/or BBB disruption in AD brain long before pro-inflammatory neurodegenerative changes and cognitive impairment occur [12,20,25-29].

## Major GI-tract Microbiome Bacterial Divisions-*Bacteroides* and *Firmicutes*

There are about ~52 major divisions of bacteria currently recognized in nature, however evolution has selected just 2 of these major divisions-*Bacteroidetes* and *Firmicutes*-to populate and form the ‘bacterial core’ the human GI-tract microbiome [30-33]. The gene set of the GI-tract microbiome has been estimated to consist of about ~2.23 × 10^7^ genes, or about ~839 times larger than the total number of genes in the human genome [1,14,32-35]. This large and diverse microbial population makes an important contribution to human metabolism, immune and endocrine health and neurobiology by contributing enzymes and metabolic factors that are not encoded by the human genome. In the healthy gut Gram-negative *Bacteroidetes* and Gram-positive *Furmicutes* coexist symbiotically, generating essential vitamins such as vitamin K, as well as most of the water-soluble B vitamins including cobalamin, folate, pyridoxine, riboflavin and thiamine, bile acids, complex sugars, polysaccharides and carbohydrates, polyphenols and cofactors, process dietary constituents such as phytochemicals, and digest dietary fiber, via the anaerobic fermentation of cellulose, lignin, and pectin into small chain fatty acids (SCFAs; chiefly acetate, propionate, and butyrate). SCFAs may enter the systemic circulation directly; many of these are precursors that play key roles in neuro-immunoendocrine regulation [36,37]. These remarkable capabilities have made these 2 phyla especially suitable to support healthy human digestion, immunology and immunoregulation, lipid metabolism, physiology, biochemistry, neurochemistry, neuroendocrinology and neurobiology [1,13,30,37-43]. Of *Bacteroidetes* and *Furmicutes*, the phyla *Bacteroidetes*, represents the largest class of obligate anaerobic Gram-negative bacteria of the human GI-tract ileum and upper large intestine where a favorable pH of 6-7.5 and obligate anaerobic conditions coexist [15,44-47]. It should be kept in mind that as for all GI-tract microbes, various strains of *Bacteroides*: (i) exhibit significant variations in abundance, stoichiometry, anaerobic and pH requirements along the ~7-meter length of the human GI-tract [48-52]; and (ii) interestingly, exhibit a basal variation in abundance, speciation and complexity amongst individual humans [1,33]. It is tempting to speculate that a certain composition and make-up of the GI-tract microbiome by all resident microbes may predispose humans to health or disease throughout the time course of life.

## Strains of the Genus *Bacteroides*-the Good, the Bad and *Bacteroides fragilis*

In contrast to their beneficial roles in the GI-tract microbiome, the genus *Bacteroides* have potential to release a remarkable and extraordinarily complex array of neurotoxins-several of the most widely recognized are lipopolysaccharide (LPS), truncated forms of LPS molecules known as lipooligosaccharides (LOS) and related enterotoxins, bacterial amyloids and small non-coding RNA (sncRNA), some of the later which resemble mammalian microRNAs (miRNAs) in their structure, molecular-genetic function and neurochemistry [7,12,21,53-58]. Strains of the abundant, GI-tract resident, obligate anaerobe bacillus *Bacteroides* are distinguished in part by their biosynthetic capabilities to synthesize and secrete unique forms of these exceptionally potent neurotoxins, the most well characterized are: (i) a *Bacteroides fragilis*-specific strain of LPS known as the glycolipid subtype BF-LPS, smaller and more compact than most known forms of Gram-negative bacterial-derived LPS; and (ii) a hydrolytic, zinc-dependent metalloproteinase toxin known as *Bacteroides fragilis* toxin (BFT) or fragilysin. Strains of *Bacteroides* that do not secrete BF-LPS and/or BFT that are generally beneficial to human health are termed non-toxigenic *B. fragilis* (NTBF), while those that do secrete LPS, BF-LPS, BFT (fragilysin) and other neurotoxic compounds are termed enterotoxigenic *B. fragilis* (ETBF; [59,60]). Recent characterization of BF-LPS and fragilysin have shown them to be: (i) unusually detrimental to the growth and health of cultured human brain cells in primary culture; (ii) toxic to transgenic murine models for age-related neurodegeneration including transgenic murine models of AD (TgAD; such as the 5 × FAD model; [61]); and (iii) probably the most pro-inflammatory microbial-derived endogenously-sourced lipoglycans and enterotoxins known [12,14, 41,42,54,57,61,62]. Indeed these secreted neurotoxic glycolipid and metalloproteinase molecules are deleterious to multiple aspects of microbiome-human brain interactions, including their ability to: (i) alter GI-tract and blood-brain barrier (BBB) structure, integrity and permeability; (ii) induce a dyshomeostasis in the systemic circulation involving biofluids such as cerebrospinal fluid (CSF), the glymphatic and meningeal lymphatic systems of the brain and CNS, and blood serum that can potentially allow these toxins to circulate throughout the body, brain neurovasculature and CNS compartments [63,64] (https://www.meliordiscovery.com/in-vivo-efficacy-models/lps-systemic-inflammation/); (iii) trigger alterations in central nervous system (CNS) and peripheral nervous system (PNS) homeostasis and equilibrium, including that of the retina and visual system [20,64,65] (https://www.meliordiscovery.com/in-vivo-efficacy-models/lps-systemic-inflammation/); (iv) induce synaptic disruption and disorganization [42,66]; and (v) promote a progressive age-related inflammatory degeneration within CNS and PNS compartments [7,12,66,67]. LPS, BF-LPS and BFT: (i) have also been found to be not only the most pro-inflammatory lipoglycans and endogenously-generated enterotoxins anywhere in the human body but also among the most barrier-disruptive, reactive oxygen species (ROS)-and inflammatory cytokine-generating toxins so far identified; and (ii) have been shown to participate in inflammation-induced alterations in synaptic plasticity and altered cognition in transgenic murine models of neurodegeneration including TgAD models [7,12,41-43,54,55,62,66,68,69].

When these highly toxic exudates of enterotoxigenic strains of *B. fragilis* escape the microbial-dense environment of the human GI-tract they can produce substantial systemic inflammatory pathology with significant mortality and morbidity. ETBF can induce and support a “smoldering” systemic infection if displaced into the bloodstream or surrounding tissue following septic disease including septicemia, trauma including chronic traumatic encephalopathy (CTE) and/or surgery [5,6,29,70]. Indeed ETBF proliferation, LPS, BF-LPS and fragilysin have long been known to associate with: (i) generalized bacteremia, anaerobic bacteremia, sepsis, systemic inflammatory distress, systemic infection, systemic inflammation and diarrheal disease; (ii) GI-tract and multiple pro-inflammatory colon and colorectal cancers [54,71]; (iii) brain and intra-abdominal abscess including intra-tumoral abscess [72,73]; (iv) cellulitis, colitis, diabetic ulcer, diarrhea, necrotizing fasciitis and peritonitis [74]; (v) the development of neurological diseases involving inflammatory neurodegeneration in part via the disruption of epithelial cell-based GI-tract barriers via cleavage of the synaptic adhesion zonula adherens protein E-cadherin, and entry of neurotoxins across biophysical barriers including the BBB [75]; and (vi) microglial-mediated innate-immune responses, detoxifying and phagocytic mechanisms, and the promotion of amyloidogenesis-these are all characteristic of the progressive inflammatory aspects of neurodegeneration [25,56,57,76-79]. Very recently LPS-induced systemic inflammation has been associated with synaptic loss and cognitive decline in multiple human neurological disorders and in transgenic animal models, and a role for LPS-mediated microglial release of pro-inflammatory cytokines such as IL-1β, based on both in vivo and primary culture studies in vitro [7,12,42,61,62, 69,79,80].

It has been further established that enterotoxigenic strains of *B. fragilis* can rapidly proliferate in the mammalian GI-tract both in the absence of adequate dietary fiber and in the presence of high-fat cholesterol diets [12,14,27,81]. This proliferation enhances the intestinal abundance of *B. fragilis* and hence the potential of this Gram-negative obligate anaerobic species to secrete its formidable array of neurotoxic exudates. Both BF-LPS and fragilysin can leak through the normally protective mucosal barriers of the GI-tract intestinal endothelium to induce substantial inflammatory pathology both systemically and after BBB transit into vulnerable CNS compartments, including, prominently the parenchyma of neocortical brain cells [27,41,42,56,57,61,70,79].

## Bacterial LPS-Proliferation by Inducers and Dietary Fiber

LPS molecules are amphipathic glycoconjugates consisting of a hydrophobic lipid domain attached to a core oligosaccharide and a distal polysaccharide with a lipid component that determines endotoxic activities and induces potent pathophysiological effects, are concentrated and shed from the outer membrane of Gram-negative bacteria [67,82]. The generation, abundance and secretion of these dynamic molecular glycolipid assemblies from Gram-negative bacteria can be stimulated by (i) proliferation of the Gram negative bacterial source itself; (ii) by neurotoxic ROS-inducing metallotoxins such as aluminum and other stressors [19,83]; and (iii) by other inducers, including those in the diet, such high fat-cholesterol (HF-C) consumption and insufficient dietary fiber, and by other unhealthy lifestyle factors [12,14,69,80,82-84]. Dietary deficiency-induced neurotoxicity of LPS are mediated in part by macrophages and microglia through the actions of tumor necrosis factor alpha (TNFα), interleukin-1β (IL-1β) and reactive oxygen species (ROS), which may be three of the most important endogenous mediators of the toxic pathophysiological effects of LPS [67,83,85]. Each Gram-negative bacillus, indeed each GI-tract resident microbe has the potential to secrete a slightly different LPS and/or neurotoxin assortment with slightly different lipid and oligosaccharide domain structures, abundances, activities, molecular properties and toxicities [12,53-55,57,67,82,86]. The modulatory effect of dietary fiber on the propagation of these microbial species and their neurotoxins remain incompletely understood. Different fiber types such as soluble versus insoluble dietary fibers appear to have different effects on the propagation of these enterotoxigenic microbes in the GI-tract and their microbe-derived neurotoxin-mediated effects, as well as on the proliferation and maintenance of GI-microbial diversity [12,14,48,81].

## Generation of *Bacteroidetes*-Derived Neurotoxins

**Figure 1:**
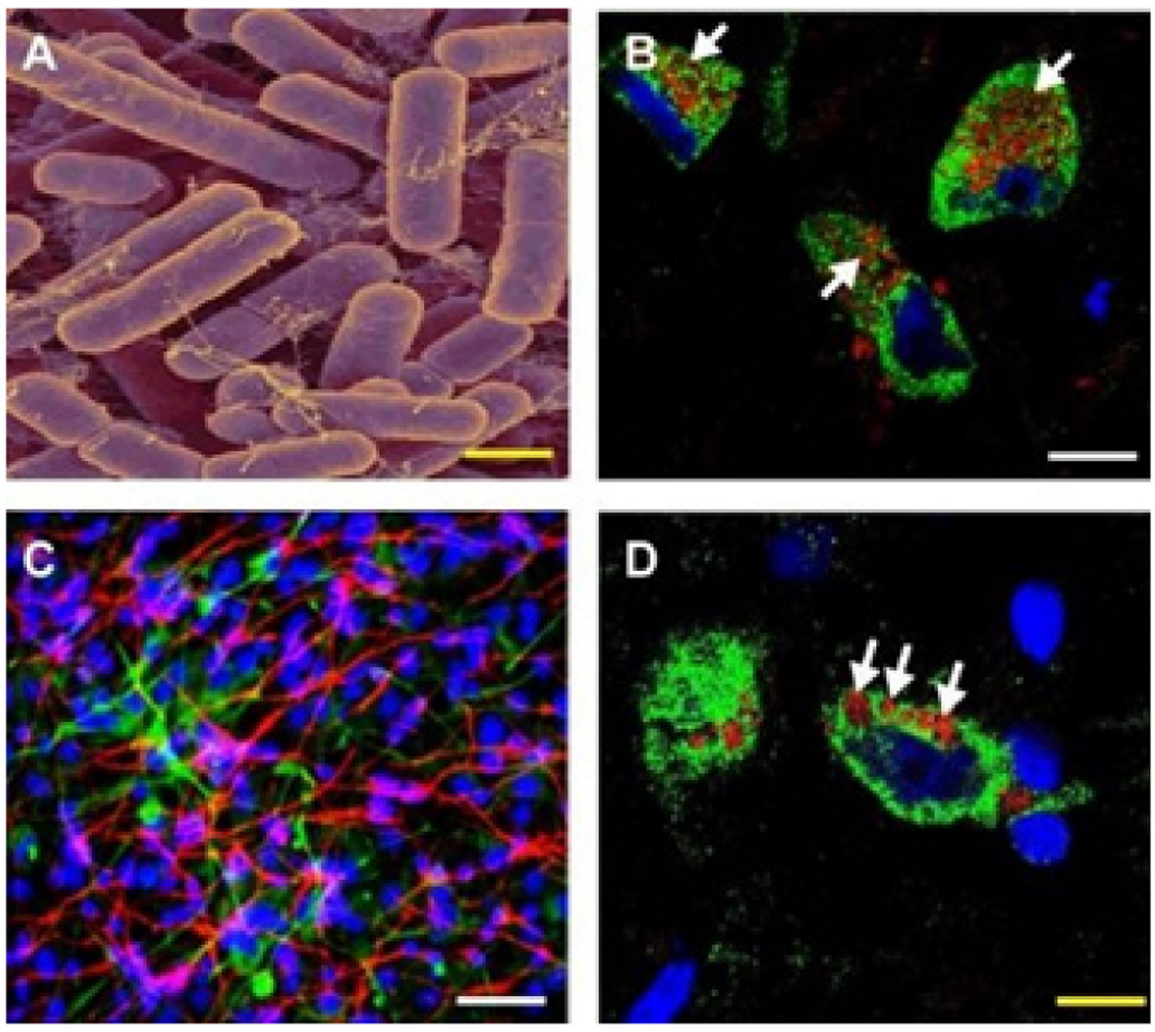
LPS generation by GI-tract abundant microbes and affinity for human brain neuronal nuclei.

As discussed above, the proliferation and absolute abundance of *B. fragilis* and *B. fragilis*-derived neurotoxins in the human GI-tract microbiome appears to be regulated in large part by the intake of dietary fiber, such that diets low in soluble fiber tend to proliferate anaerobic Gram-negative bacterial species and their exudates from the genus *Bacteroides* and others [7,12,14,15, 55,81, 87-90]. Post ingestion of dietary fibers into the GI-tract lead to their rapid catabolization into short-chain fatty acids (SCFAs), volatile fatty acids, polysaccharides and other digested components in part through the biosynthetic capability of multiple GI-tract microbial species including *B. fragilis* [54,71,87]. As described above, when *B. fragilis* or *B. fragilis*-derived neurotoxins escape the microbe-dense environment of the GI-tract they can induce substantial systemic inflammatory pathology with significant sickness, morbidity and mortality [7,12, 56,76,91]. In mammalian animal models multiple species of *Bacteroidetes* have been shown to propagate in mammalian models fed high fat-cholesterol (HFC) diets deprived of sufficient intake of dietary fiber suggesting that dietary fiber may have a significant role in regulating the abundance, complexity, speciation and and stoichiometry of microbial constituents of the GI-tract microbiome and their secretory products, including those of *B. fragilis* [12,14,81,91,92]. It is important to point out that: (i) that many other types of GI-tract resident Gram negative bacteria and/or other microbes also secrete a bewildering complexity of LPS and enterotoxins that are highly complex in their composition and may be synergistic in their neurotoxicity; (ii) that all of these broad spectrum of neurotoxins are endogenously sourced; and (iii) that they are constantly available with variable abundance throughout the lifespan of the host organism [7,12,54,81,87, 91-93] (Figure 1).

A-Obligate anaerobic Gram-negative bacilli including the murine and human gastrointestinal (GI)-tract abundant *Bacteroides fragilis* are capable of releasing a broad spectrum of highly neurotoxic, pro-inflammatory and potentially pathogenic molecules; these neurotoxins comprise at least six classes of secreted moleculartoxins and include lipopolysaccharide (LPS), lipooligosacahride (LOS; small truncated forms of LPS), endotoxins, exotoxins, bacterial-derived amyloids, and small non-coding RNAs (sncRNA). Each different species of GI-tract resident microbe appears to have its own repertoire of species-specific neurotoxins. Enterotoxigenic *Bacteroides fragilis* cultures have been extracted to generate extracts enriched in a *B. fragilis*-specific LPS known as BF-LPS and other neurotoxins such as *B. fragilis* toxin (BFT; also known as fragilysin [25,86]; the human GI-tract-abundant *B. fragilis* secretes both BF-LPS and BFT which have been recently shown to be strongly pro-inflammatory and extremely neurotoxic toward human neuronal-glial (HNG) cells in primary co-culture [57]. While the phyla Bacteriodetes (~20% of all GI-tract bacteria), *Firmicutes* (~80% of all GI tract bacteria), *Actinobacteria, Proteobacteria,* and *Verrumicrobia* (together, typically about ~1% of all GI-tract bacteria), are the most common microbes in the human GI-tract microbiome it should be kept in mind that other microbes including fungus, protozoa, viruses, and other commensal microorganisms may also contribute neurotoxic exudates which can be toxic and detrimental to the homeostasis of CNS neurons. Micrograph of *B. fragilis* shown; yellow filamentous strands in panel A stain as LPS; original photo courtesy of Rosa Rubicondior; (http://rosarubicondior.blogspot. com/2014/11/evolving-cooperation-but-for-who-or-what.html); yellow scale bar (lower right)=2 μm; B-Association of lipopolysaccharide (LPS; red stain; λmax=690 nm) with the neuronal cytoplasm and the periphery of neuronal nuclei in AD neocortex; NeuN (neuron-specific green stain; λmax=520 nm) and DAPI (blue stain; λmax=470 nm); human superior temporal lobe AD neocortex (Brodmann A22); note affinity and polarized association of LPS for the neuronal nuclear envelope (white arrows; [93-97]); white scale bar (lower right)=20 μm; C-Control human neuronal-glial (HNG) cells in primary culture stained with antibody to glial fibrillary acidic protein (GFAP), a glial-specific cytoplasmic marker (green fluorescence; λmax=556 nm); an antibody to the neuron-specific cytoplasmic marker βTUBIII, (red; λmax=702 nm), and with Hoescht 33258 to highlight the morphological features of both glial- and neuronal-cell nuclei (blue; λmax=461 nm; 2 weeks in culture; HNG cells are about ~60% neurons (red) and about ~40% astroglial (green); 20x magnification); human primary neuronal and glial “support” cell co-cultures are utilized because human neuronal cells do not culture well by themselves [25]; note large nuclear area, relative to both glial or neuronal cytoplasmic area, indicative of high levels of transcriptional activity; white scale bar (lower right)=20 μm; D-HNG (transplantation grade) cells in primary co-culture were used to study the dynamics of LPS association with neurons [4,62,47,92]. Dark blue stained spherical and oval bodies are DAPI-stained non-neuronal nuclei from astroglia; white arrows indicate punctate and perinuclear clustering of LPS and LPS affinity for the nuclear envelope as has been previously reported [25,26,30,46,47,89]; both in vivo analysis in AD brain (Figure 1B) and in vitro experiments using HNG cells in primary tissue culture (Figure 1D) show affinity of LPS for neuronal nuclei; yellow scale bar (lower right)=20 μm.

## *Bacteroidetes* Neurotoxins, their Mechanism of Action and Alzheimer’s Disease (AD)

Over the last five years LPS and/or BF-LPS have been shown: (i) to associate with the periphery of neuronal nuclei in sporadic Alzheimer’s disease (AD) brain [3,83,94]; (ii) to promote the generation of reactive oxygen species (ROS), the inflammatory transcription factor NF-kB (p50/p65 complex) in human neuronal-glial cells in primary-culture [7,12,57,83]; (iii) to efficiently enter neurons with the assistance of Aβ42 peptides, perhaps as a consequence of Aβ42 peptides’ ‘pore-forming’ capabilities [95,96]; and (iv) to strongly induce the upregulation of the pro-inflammatory transcription factor NF-kB (p50/p65 complex) and transcription of a selective family of pro-inflammatory NF-kB-sensitive microRNAs (miRNAs) that include miRNA-9, miRNA-34a, miRNA-125b, miRNA-146a, and miRNA-155 [24,57,62, 83,97,98]. These miRNAs in turn ultimately bind to the 3’-untranslated region (3’-UTR) of multiple target messenger RNAs (mRNAs) in brain cells and thereby reduce their expression post-transcriptionally. Down-regulated mRNAs include those encoding complement factor-H (CFH) of the innate-immune system [83,99], an SH3-proline-rich multi-domain-scaffolding protein of the postsynaptic density (SHANK3; [98]); and the triggering receptor expressed in myeloid/microglial cells (TREM2) surface glycoprotein immune receptor [4,100,101]. The down-regulation and/or loss-of-function mutations of CFH, SHANK3 and/or TREM2 expression are also observed in AD brain and/or in transgenic murine models of AD (TgAD; [83,100,101]). Hence, an extremely toxic LPS normally confined to the GI-tract is capable of driving a NF-kB-miRNA-mediated deficiency and disruption in gene expression that contributes to alterations in synaptic-architecture and synaptic-deficits, amyloidogenesis, innate-immune defects, and progressive inflammatory signaling, all of which are characteristic of AD-type neuropathology and multiple states of brain cell degeneration. Hence BF-LPS as well as ‘generic LPS glycolipids’ from other Gram negative GI-tract resident bacilli represent an important pathogenic initiator component of an NF-kB-miRNA-mRNA signaling program that has potential to down regulate specific gene expression patterns known to be required for normal CNS homeostasis, and hence contribute progressively to AD-type neurodegenerative change.

## LPS Entry into CNS Neurons-Facilitation by Aβ42 Peptides

**Figure 2:**
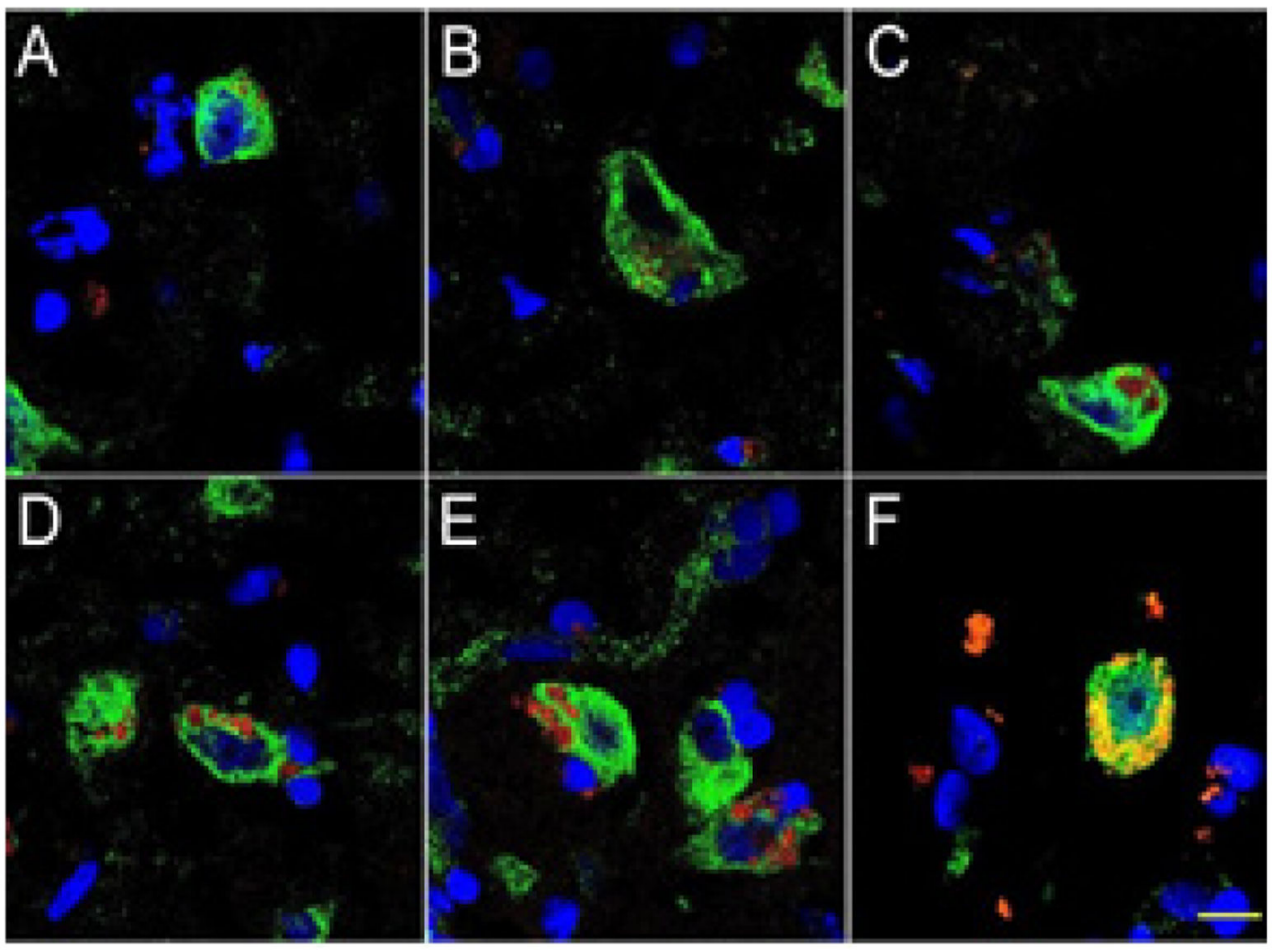
Details of the association of LPS with neuronal neocortical nuclei and progressive association and envelopment of AD-affected neocortical neuronal nuclei by LPS.

Recent reports have indicated a remarkably high affinity of microbiome-derived LPS for human brain neuronal nuclear membranes resulting in the complete envelopment of neuronal nuclei in AD brain neocortex, especially in the middle-to later-stages of AD (Figure 2). This ‘encapsulation of neuronal nuclei’ in AD brain by LPS has a disruptive effect on the generation of important neuron-specific transcription products that include the presynaptic phosphoprotein synapsin-2 (SYN-II) that associates with the cytoplasmic surface of synaptic vesicles, and the neurofilament light (NF-L) chain filament protein that maintains the overall shape and cytoarchitecture of the neuron [42,66,97,98,102].

Human superior temporal lobe AD neocortex (Brodmann A22) from CDR 1.0, (panel A, B); CDR 2.0 (panel C, D) and CDR 3.0 AD brains (panels E, F); see also [25,26]; https://knightadrc.wustl.edu/cdr/cdr.htm; (red stain; λmax=690 nm), DAPI (blue nuclear stain; λ max=470 nm) and NeuN (neuron-specific green stain; λmax=520 nm); LPS staining (red) was subjected to co-localization analysis with the neuronal marker NeuN (green) and/or nuclear marker (blue). In middle-to-late stages of AD approximately 30-50% of all neuronal neocortical nuclei were fully or partially encapsulated by LPS; complete envelopment of neuronal nuclei by LPS has deleterious effects on the transcription of neuron-specific genes including neurofilament-light (NF-L) and synapsin (SYN-2) gene expression [7,79]; interestingly the enterotoxigenic *B. fragilis*-specific form of LPS known as BF-LPS is is a more compact form of LPS and differs from the LPS of other Gram-negative bacteria; Aβ42 peptides forming channels in neuronal nuclear membrane may allow LPS and/or BF-LPS access to genetic material with detrimental AD-relevant effects; [7,25,26]; scale bar in panel F is ~20 um and is representative for all 6 panels.

**Figure 3:**
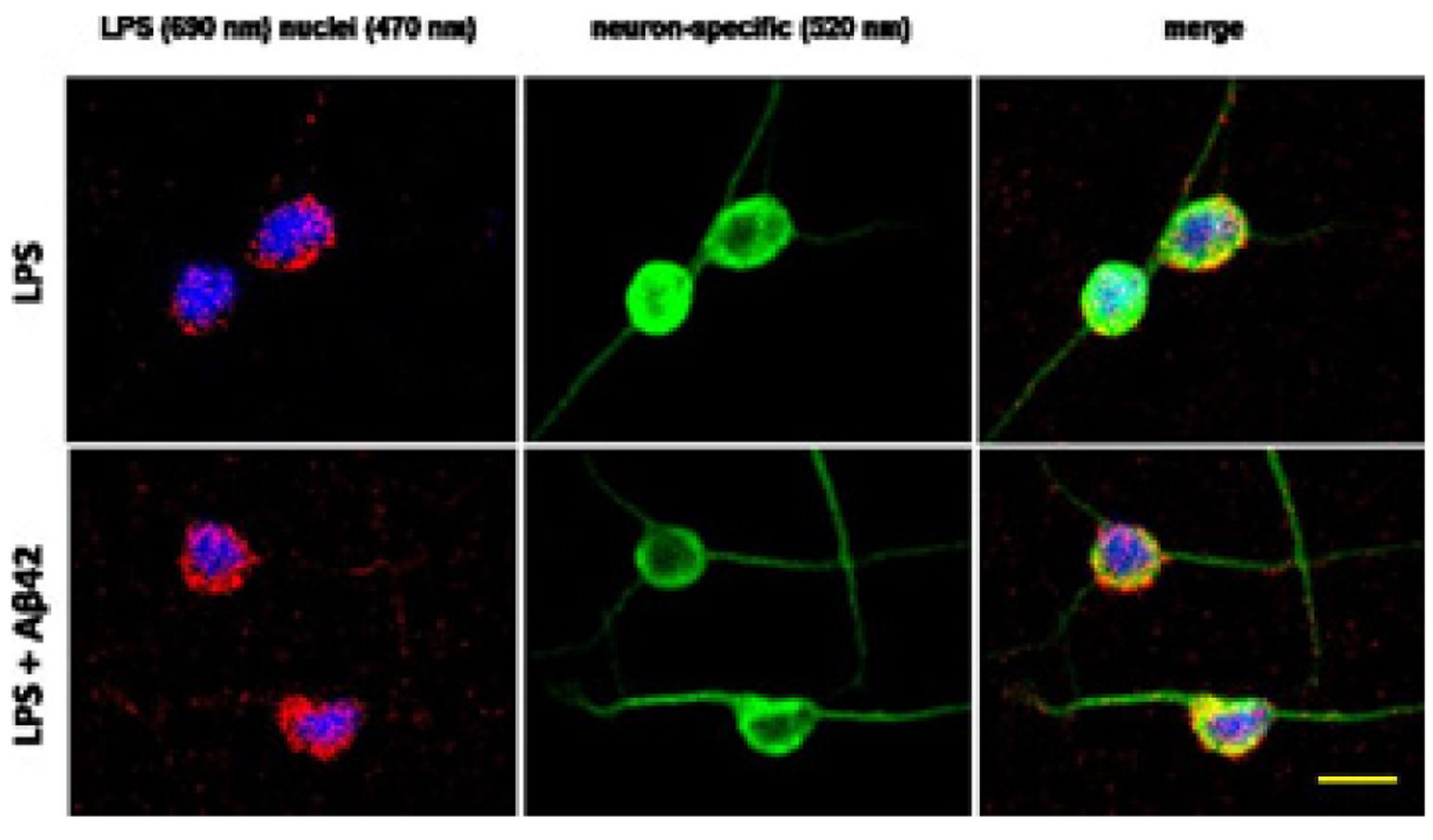
Aβ42 peptides facilitate association and entry of LPS into neurons.

Probably the major biomarker for AD is the excessive abundance of amyloid beta (Aβ) peptides, both free and aggregated into senile plaque deposits in the parenchyma of the AD brain. Almost 30 years ago it was discovered that Aβ peptides support the formation of different types of atypical heterogeneous ionic pores and/or ion conducting channels spanning the lipid bilayer of the plasma membrane and that pore formation is linked to the pathogenicity and development of AD [95,103,104]. Since then these findings have been repeatedly supported by multiple independent reports regarding the ability of Aβ42 peptides [2 hydrophobic amino acid residues (isoleucine and alanine) longer at the C-terminal than the Aβ40 peptide] to form ~2.4-3.0 nm diameter pores through lipid bilayer membranes through which LPS and other neurotoxins may translocate across the cellular and/or nuclear plasma membrane [95,104-108]. Note that LPS has a MW ~10-20 kDa as an SDS-dissociated monomer with highly variable geometry and the basic monomeric shape of a flexible cylindrical molecule approximately 2.4 nm in diameter and 9.6 nm in length; the unique LPS of *B. fragilis* (BF-LPS) is significantly smaller than ‘generic LPS’ from other Gram negative bacteria such as *Escherichia coli*. There is evidence that Aβ42 peptides: (i) may facilitate the entry of both LPS through neuronal plasma and neuronal nuclear membranes by a localized membrane disruption or disorganization of the lipid bilayer [95,104,108]: (ii) may allow LPS transit into the cytoplasm or nucleoplasm through the lipid bilayer by pore formation [95,108]; and (iii) as a consequence of their entry into the cytoplasm or nucleoplasm both Aβ42 peptides and/or LPS may interact with nucleosomes, nucleoproteins, nucleic acids and other intra-nuclear structures in altering chromatin organization, disrupting transcription and/or modifying genetic or epigenetic gene regulation in the context of the development of disease [95,104,108] (Figure 3).

There is accumulating evidence that Aβ42 peptides: (i) facilitate both the association and entry of LPS through neuronal plasma and neuronal nuclear membranes, an effect not observed with GFAP-staining (astrocytic) brain cells [95,108,104]: (ii) allow LPS entry into the cytoplasm or nucleoplasm through a local disruption or disorganization of the lipid bilayer or by pore formation [7,25,26,104]; and (iii) as a consequence of their entry into the cytoplasm or nucleoplasm both Aβ42 peptides and/or LPS may interact with nucleosomes, nucleoproteins, nucleic acids and other intra-nuclear structures in altering chromatin organization, disrupting neuron-specific transcription and/or modifying genetic or epigenetic gene regulation in the context of the progressive development of neurological disease [7,95,104,108]; as in Figure 2 HNG cells (see text) were differentiated from human neural progenitor cells (HNPCs) and culture D for 2 weeks before being exposed to 50 nM LPS for 36 hr in the presence or absence of 10 nM Aβ42 peptide; staining: red stain; λmax=690 nm; DAPI (blue nuclear stain) λmax=470 nm and NeuN (neuron-specific green stain) λmax=520 nm); LPS staining (red) was subjected to co-localization analysis with the neuronal marker NeuN (green) and/or nuclear marker (blue); neuronal nuclei are approximately 20 um in diameter; scale bar in rightmost lower panel is ~20 um and is representative for all 6 panels [7,25,47,95].

## The GI-Tract Microbiome at the Cessation of Life-the Thanatomicrobiome

There are enormous biological, biophysical, immunological, physiological and bioenergetic efforts in keeping the GI-tract microbiome contained within GI-tract compartments and from expansion beyond its normal niche, and our understanding of this comes in part from analysis of the human microbiome that evolves at the time of death. At the cessation of life the generation of energy-containing nucleotide triphosphates ceases, nucleotide triphosphate stores such as those of ATP are rapidly depleted, circulatory systems involving the CSF, lymph and glymphatic system and blood flow ease and eventually terminate, the immune system falters and microbes proliferate throughout the body. In the healthy adult for example, internal organs that include the spleen, liver, heart, and brain are generally devoid of enterotoxigenic microbes such as ETBF because the innate-immune system or other microbial components keeps them in check. Relatively little is known on exactly what happens to the human GI-tract microbiome when the human host dies, and the process of human tissue decomposition is exceedingly complex. The concept of the thanatomicrobiome (thanatos, Greek for death; sometimes referred to as the ‘epinecrotic microbiome’ or ‘necrobiome’), defined as the composition, organization and expansion of the normal GI-tract microbiome and other microbial communities following cessation of all life and neurological activity, in part after the observation of a zero line electroencephalogram (ZLEEG) in multiple brain regions [109,110]. The community of microbial species associated with the decaying corpse remains a relatively recently appreciated area of scientific investigation from multiple neurological, forensic and neuropathological aspects. Emerging studies indicate that this series of catabolic events appears to begin in the ileocecal area of the GI-tract, spreading to the liver and spleen, continuing to the heart and brain [111-114]. Ongoing work from temporal studies on the thanatomicrobiome across a defined series of PMIs further indicate: (i) there is a constant struggle to contain GI-tract microbiome integrity and regulate specific bacterial abundance and complexity right up to the cessation of all life activity [111,112]; (ii) that the majority of the microbes within the human body and those which propagate most rapidly at the time of death are the obligate anaerobes (such as *B. fragilis)* that begin to non-randomly proliferate from deeper regions of the GI-tract continuing throughout the human organs over the PMI [112,113]; and (iii) that comprehensive knowledge of the number and abundance of each organ’s microbial signature is critical to forensic microbiologists, neuropathologists, human microbiome and post-mortem tissue researchers as a new source of data for estimating microbial complexity and evolution over the PMI [1-3,112-114]. These data combined with DNA-and RNA-based nucleic acid sequencing and bioinformatics are also essential in aiding researchers who use post-mortem tissues in their research work, in forensic criminology, and the study of microbial speciation, complexity and microbiome-host genetics in the later stages of life. The very rapid post-mortem appearance of multiple genus/species of microbes in the brain and CNS at the time of death lends credence to the concept that these microbes would not have enough time to transit from the microbiome of the GI-tract to the brain, especially when systemic circulation of all types have essentially ceased, and suggests that microbes are already present within the confines of the brain and CNS compartments [7,12,113,114].

## A Brain Microbiome?

While multiple research reports provide abundant evidence that GI-tract microbiome-derived neurotoxins such as LPS, BF-LPS, fragilysin and other microbial-sourced toxins are fully capable of disrupting, damaging and crossing biophysical barriers there is also the possibility that the brain and CNS possesses its own microbiome. Indeed multiple CNS compartments such as the eye, derived from the neural crest cells: (i) appear to harbor their own microbiome; and (ii) there is an interesting association of dysbiotic changes in the GI-tract microbiome with extra-intestinal diseases such as ocular diseases include bacterial keratitis, fungal keratitis, uveitis, age-related macular degeneration and ocular mucosal diseases [115-117]. Some of the strongest support for a brain microbiome comes from studies on the thanatomicrobiome that evolves following cessation of all life-relevant biological, biochemical, neurochemical and neurophysiological activities [25,111-113, 118]. In a healthy adult, most internal organs such as the spleen, liver, heart, and brain are generally devoid of enterotoxigenic microbes because the innate-immune system and/or other microbial components keeps them in check, however, certain microbial species rapidly appear in the thanatomicrobiome of the brain post-mortem, faster than transport would allow these bacteria transit from the GI-tract into CNS compartments, suggesting that some type of microbial community already populates the brain and CNS. Widespread independent reports documenting: (i) the presence of microbes and/or their secreted neurotoxins, especially viral-and bacterial-derived neurotoxins and related components deep within the brain parenchyma, cytoplasm and nucleoplasm either free, plasma-membrane-bound or vesicle-bound further lend support to this intriguing idea; and (ii) 16S ribosomal RNA (rRNA) next generation sequencing analysis have repeatedly shown bacterial and microbial nucleic acids in post-mortem brain from AD patients [4-6,13,24,26,57,74,94,113,118,119].

Related to the idea of a brain microbiome is the assumption of the ‘privileged immunological status’ of the CNS also continues to be questioned, particularly in terms of inflammatory neurodegenerative diseases such as AD, as both microbial-derived nucleic acid sequences and/or noxious exudates representative of GI-tract Gram-negative bacteria and neurotropic viruses such as herpes simplex 1 (HSV-1) have found to be resident within CNS compartments. These compartments include, prominently, anatomical regions of the CNS involved in inflammatory and pathological signaling and neuro-immune disruptions that characterize the AD process [25,26,57,94,118,120-122]. For example, LPS has been recently localized to the same anatomical regions involved in AD-type neuropathology to levels of greater than 7-fold over control in the temporal lobe neocortex and at least ~20-to-25-fold over controls in the hippocampus, suggesting that GI-tract microbiome-derived LPS may be an important initiator and/or significant contributor to inflammatory degeneration in the AD affected CNS over a background of aging [7,12,25,26,47,118].

## Unanswered Questions

A number of unanswered questions remain regarding GI-tract microbiome-derived microbial neurotoxins and their contribution to human neurological disease and more specifically to the development of progressive inflammatory neurodegeneration such as that observed during the onset, propagation and course of sporadic AD. These include: (i) the major risk factor for AD is age-is there a progressive age-related contribution of a single neurotoxin or cocktail of neurotoxins over the human lifespan that cause a slowly developing life-long neuronal damage to the point where there is sufficient brain cell damage or dropout to cause AD-type change and a failure in neuronal signaling and cognition as humans age?; (ii) related to the previous point-does life-long exposure to specific GI-tract derived infectious agents, their secreted neurotoxins and/or environmental toxins in our diet and/or environment predispose the brain and CNS to the development of AD at a later age?;(iii) what combinations of microbes and/or bacterial-and/or viral-based neurotoxins and perhaps other microbiome-derived toxins are the harmful to the CNS and PNS and which are the most efficient in inducing a progressive pro-inflammatory neurodegeneration such as that observed in AD?; (iv) are other potentially pathogenic transcription factors in addition to NFkB (p50/p65) and other pro-inflammatory pathology-associated microRNAs besides miRNA-9, miRNA-34a, miRNA-125b, miRNA-146a, and miRNA-155 involved in driving and supporting progressive neurodegenerative AD-type change and cognitive decline?; (v) do microbiome-derived neurotoxins or polymicrobial infections exhibit synergism in their toxicities toward neural cells of the PNS and CNS?; (vi) do prebiotics, nucleic acids, carbohydrates and/or specialized plant-based dietary fibers that nourish beneficial microbes already in the GI-tract, or probiotics, consisting of beneficial “health-and homeostasis-promoting” microbes have any role in AD onset or progression?; (vii) would it be possible to devise prebiotic, probiotic, anti-neurotoxin, anti-NF-kB (p50/p65), anti-microRNA, dietary fiber manipulation or related approaches, or combinations of these therapeutic strategies to benefit the clinical management of AD once the disease has already taken hold?; (viii) does the brain and CNS possess its own microbiome, and if so what is its composition, structure and function in neurological health and disease?; (ix) do specific combinations in individuals of species or stoichiometries of GI-tract microbes that include aerobic or anaerobic, Gram negative or Gram-positive bacteria, archaebacteria, fungi, protozoa, viruses and other microorganisms and/or their secreted neurotoxins favor the development of systemic inflammation, inflammatory neurodegeneration or other types of human disease?; (x) do multiple whole-body microbial infections earlier in life predispose to the development of AD at a later date?: and (xi) perhaps most importantly, could it be possible to tailor a life-long dietary intake and nutritional management strategy, perhaps along the lines of a ‘personalized medicine approach’, that creates a GI-tract microbiome that is conducive to lowered abundances of Gram-negative-derived neurotoxins and a dietary intake that effectively minimizes the risk of CNS-based age-related neuroinflammatory diseases such as AD?

## Discussion and Summary

The human GI-tract microbiome remains a vast and understudied repository of microbial species, and prokaryotes and their genes considerably outnumber the total number of host cells and host genes contained in the human genome [1,7,12,14,32-35,57]. This large and diverse microbial community makes an important contribution to human metabolism, innate-immune health and neurobiology by contributing a considerable number of enzymes and metabolic factors that are not encoded by the human genome. This genus-, species-and genetically-diverse microbial population: (i) forms a complex, dynamic, and highly interactive microbial community that plays major roles in digestion, nutrition, inflammation, growth, immunity against foreign pathogens and neurological disorders that include neurodegeneration [5,15,45,80]; (ii) has symbiotic associations and interactions with the host indispensable for human health and homeostatic physiological functions [13,22,38,123]; and (iii) exhibits fluctuations in individual abundance, speciation, stoichiometry and complexity in response to developmental stage, dietary factors, GI-tract disturbances, aging, and disease [16,38,46]. An imbalance in microbial populations in the GI-tract of AD patients has been reported in several studies, and this dysbiosis very likely increases the abundance of GI-tract microbiome-derived molecules that may be detrimental to the human metabolism and neurological health. The high variability in microbial complexity however, even amongst healthy individuals has made it difficult to link specific microbial abundance patterns with age-related neurological disease such as AD [15,33,43,118]. There is strengthening evidence that: (i) the ability of GI-tract microbiome-resident bacteria to influence neuro-immune functions well beyond the confines of the GI-tract; (ii) that changes are communicated to the brain and CNS through a GI-tract-CNS network, sometimes called the ‘gut-brain-axis’, in part via small signaling molecules such as SCFAs or other chemical messenger signaling systems; (iii) that microbial components of the GI-tract microbiome such as BF-LPS and/or the microbes themselves can transverse biophysical barriers without too much difficulty and contribute to AD-type change; and (iv) that specific GI-tract microbiome-derived neurotoxins have a strong pathological role in eliciting an up-regulation of ROS and pro-inflammatory NF-kB-miRNA-directed gene expression that is both AD-relevant and propagates the AD process [7,12,15,16,27]. Established pathways of gut-brain axis communication currently include the autonomic nervous system (ANS), the enteric nervous system (ENS), the neuroendocrine system, the immune system, the systemic circulation and vesicular trafficking [9,13,15,43,124-127]. Surprisingly, neuronal signaling pathways along the bidirectional gut-brain axis remain an understudied research area despite their important roles: (i) in coordinating metabolic-, nutritive-and neurobiological-functions, and (ii) in their functional disruption in chronic diseases such as metabolic syndrome, diabetes, obesity, anxiety, autoimmune-disease and stress-induced neuropsychiatric disease and neurodegenerative brain diseases such as AD [7,12,15,37,43,119,117,128-132].

## Conclusion

Lastly, an intriguing thanatomicrobiome develops in the postmortem human body and lack of metabolic, bioenergetic, immune and neuroendocrine systems facilitate microbial succession in the GI-tract, systemic circulation, brain and CNS compartments. The initial verification of prokaryotic 16S rRNA sequences within the AD brain using next generation sequencing, the appearance of LPS and other microbial-derived neurotoxins in brain tissues in AD, the occurrence of GI-tract-derived microbial species within the brain and relatively rapid appearance of a novel thanatomicrobiome in the brain after death distant from the abundant microbial species of the GI-tract microbiome suggests that a poorly understood and insufficiently characterized brain microbiome is most likely already there.

## Data Availability

The original contributions presented in the study are included in the manuscript text; further inquiries can be directed to the corresponding author/s.
